# Sulfate-Reducing Bacteria: Biofilm Formation and Corrosive Activity in Endodontic Files

**DOI:** 10.1155/2018/8303450

**Published:** 2018-05-10

**Authors:** Fabiano Luiz Heggendorn, Aline Guerra Manssour Fraga, Dennis de Carvalho Ferreira, Lucio Souza Gonçalves, Viviane de Oliveira Freitas Lione, Márcia Teresa Soares Lutterbach

**Affiliations:** ^1^School of Pharmacy, Federal University of Rio de Janeiro Pharmaceutical Laboratory Bioassays, Rio de Janeiro, RJ, Brazil; ^2^Laboratory of Biocorrosion and Biodegradation, National Institute of Technology, Rio de Janeiro, RJ, Brazil; ^3^Laboratory of Organic Synthesis and Medical Chemistry, School of Pharmacy, Federal University of Rio de Janeiro, Rio de Janeiro, RJ, Brazil; ^4^Faculty of Dentistry, Estácio de Sá University, Rio de Janeiro, RJ, Brazil; ^5^Veiga de Almeida University, Rio de Janeiro, RJ, Brazil

## Abstract

**Aim:**

This study describes the biofilm formation and the corrosive capacity of sulfate-reducing bacteria (SRB) on the metallic structure of used endodontic files.

**Methods:**

Sulfate-reducing bacteria (SRB) (*Desulfovibrio desulfuricans* oral and *Desulfovibrio fairfieldensis* or *D. desulfuricans* environmental) were inoculated into the culture media (Postgate C culture medium or modified Postgate E culture medium). The biocorrosive potential of these bacteria will be an important component of a biopharmaceutical under development called BACCOR. Afterwards, four used endodontic files (UEFs) were separately inoculated into a specific culture media for 445 days at 30°C in an incubator. The four UEFs were placed in a scanning electron microscope (SEM) and analyzed by the energy-dispersive X-ray spectrometry (EDS).

**Results:**

The confocal laser scanning microscopic images indicate the presence of biofilm in the four samples. The SEM and SEM-EDS revealed the presence of rough, irregular structures adhering along the metallic surface of the used endodontic files, suggesting a mature calcified biofilm with a high concentration of Ca, P, C, and S.

**Conclusion:**

The formation of SRB biofilms on used endodontic files shows characteristics that may contribute to the biocorrosion of these files, and the results may also provide complementary data for a biopharmaceutical, which is still under development to assist in the removal of fractured endodontic files inside root channels.

## 1. Introduction

Manual endodontic files are manufactured with austenitic stainless-steel alloys and used in root canal treatments to remove organic substrates, debris, and microorganisms [1, 2]. These instruments are relatively resistant to corrosion due to the chromium content in their microstructure that forms a passive film of chromium oxide. However, when this film is worn out, corrosion can set in, resulting in the loss of cutting efficiency and an increased risk of the file fracturing inside the root canal [1].

Parallel to this type of corrosion (inorganic), there is biocorrosion which is due to the corrosive action of microorganisms such as sulfate-reducing bacteria (SRB), which actively participate in the corrosive process by initiating or accelerating the electrochemical reaction of metal dissolution [3]. SRB can be found in the environment, soil, freshwater, and salty marshes or in the human body, mainly in the intestinal flora, where the species *Desulfovibrio desulfuricans* are often detected [1, 4–8].

Biofilms formed by SRB can modify the metal/solution interface to induce, accelerate, and/or inhibit the anodic or cathodic process that controls corrosion. These biofilms are formed by a gelatinous matrix with high water content (approximately 95%) where the metabolite products and microorganisms are in suspension. These cells, which are immobilized on a substrate, are included in an organic matrix of extracellular polymers which is known as extracellular polymeric material (EPM). This material agglutinates and involves the SRB, providing a protection against external agents [3, 5, 9].

The aim of this study is to describe the biofilm formation and the corrosive capacity of sulfate-reducing bacteria (SRB) on the metallic structure of used endodontic files. Understanding the corrosive capacity of these bacteria is important because the biopharmaceutical BACCOR is based on the biocorrosive potential of these bacteria. This biopharmaceutical is still under development to aid in the removal of fractured endodontic files from root canals. Previous studies have demonstrated that *D. desulfuricans* and *D. fairfieldensis* are capable of promoting biocorrosion of endodontic files [10], and cytotoxicity tests have shown that the inoculation vehicle (used in this work) is biocompatible [11].

## 2. Materials and Methods

### 2.1. Culture Medium

The modified Postgate E medium and Postgate C medium with the addition of 7.0 g/l agar-agar, indicated for the growth and isolation of SRB [5], were prepared for the assays.

### 2.2. Sample Preparation and Evaluation of Cell Growth

The bacterial strains (SRB) were inoculated into the culture media and subsequently the sterile used endodontic files (UEFs) were inserted through the rubber cap and remained in a stable vertical position throughout the assay period. The four used endodontic files in this evaluation had an unknown clinical history and were collected from a private practice. Each of these files was incubated in a specific culture media for 445 days at 30°C in an incubator, as shown in Table 1. After 30 days of inoculation and at the end of the experiment period (445 days), the cultures with the endodontic files were visually evaluated for SRB growth (Table 1).

After the 445-day incubation period, the samples were removed with care in order not to destroy the possible biofilm formed on the metal surface of the file. Immediately, 1.0 ml of the culture of each sample was replicated in modified Postgate E culture medium to assess cell viability, and the pH was measured using Universal Indicator Strips (pH 0–14; Merck).

The UEF-3 sample was submitted to immediate confocal laser scanning microscopy (CLSM). The other endodontic files were immersed in a fresh culture medium (the same as the previous one) in order to maintain the biofilm hydrated, sealed, and incubated at 30°C until needed (Table 2). The UEF-4 file was left immersed in the fresh medium for extra seven days, and the UEF-1 and -5 files for an extra 14 days. At the end of the reincubation period, the samples were removed from the culture medium to record the pH, and a replica in modified Postgate E medium was prepared from each sample (Table 2).

### 2.3. Confocal Laser Scanning Microscopy

A confocal laser scanning microscope (CLSM) (Zeiss LSM 710/LSM 710 NLO and Confocal 3) was used to analyze any biofilm formation on the endodontic files, and the images obtained were analyzed and processed with ZEN 2009 software (Zeiss). The Live/Dead® kit was used as per the manufacturer's instructions (FilmTracerTM Live/Dead Biofilm Viability Kits, Invitrogen™) with a fluorophore that is able to identify living and dead cells in a mixed population. The fluorophore was composed of SYTO® 9, which marks living and dead cells a fluorescent green, and propidium iodide (PI), which marks the dead cells red, penetrating only bacteria with damaged membranes, overlapping the SYTO 9. The fluorophore was prepared as per the manufacturer's instructions, and then each used endodontic file was immersed for a 15-minute incubation period in a dark environment, after which the files were examined in the CLSM.

After the UEF analysis in the CLSM, the files were immersed in alcohol 70 (ethyl alcohol hydrated 70° INPM) followed by washing in distilled water.

### 2.4. Scanning Electron Microscopy

A scanning electron microscope (SEM-FEI-Inspect-S50) was used to visualize the metallic surface of the endodontic files after SRB growth. The SE, BSE, and EDS analytical modes were applied.

The SE (secondary electron) mode provides high-resolution images where the contrast in the image is given by the relief of the sample. The BSE (backscattered electron mode) method provides images of the composition, with contrast as a function of the atomic number of the elements present on the surface of the sample.

The energy-dispersive X-ray spectrometry (EDS) identifies the chemical elements by mapping the spatial distribution of these elements generating composite X-ray maps (X-ray mapping) or spot analyses and a spectrum of energy demonstrating the relative number of chemical elements present, with a penetration power of 1 *μ*m of the electron beam, thus determining qualitatively and quantitatively the elements present in the sample [12, 13].

The control sample was a new Kerr No. 30, 25 mm endodontic file (K-File 25 mm, 030; Dentsply Ind. and Com. Ltda.; Maillefer Instruments, Switzerland; LOT: 8226850; Ref.: A012D02503012).

## 3. Results

### 3.1. Cell Viability and Confocal Laser Scanning Microscopy

The pH of all the culture samples was 7. The images from the CLSM indicated the presence of biofilm in all four samples. In addition, the cell viability of the cultures was checked by the replicas in the Modified Postgate E medium (Table 3) for correlation with the microscopic analyses.

**Figure 1:**
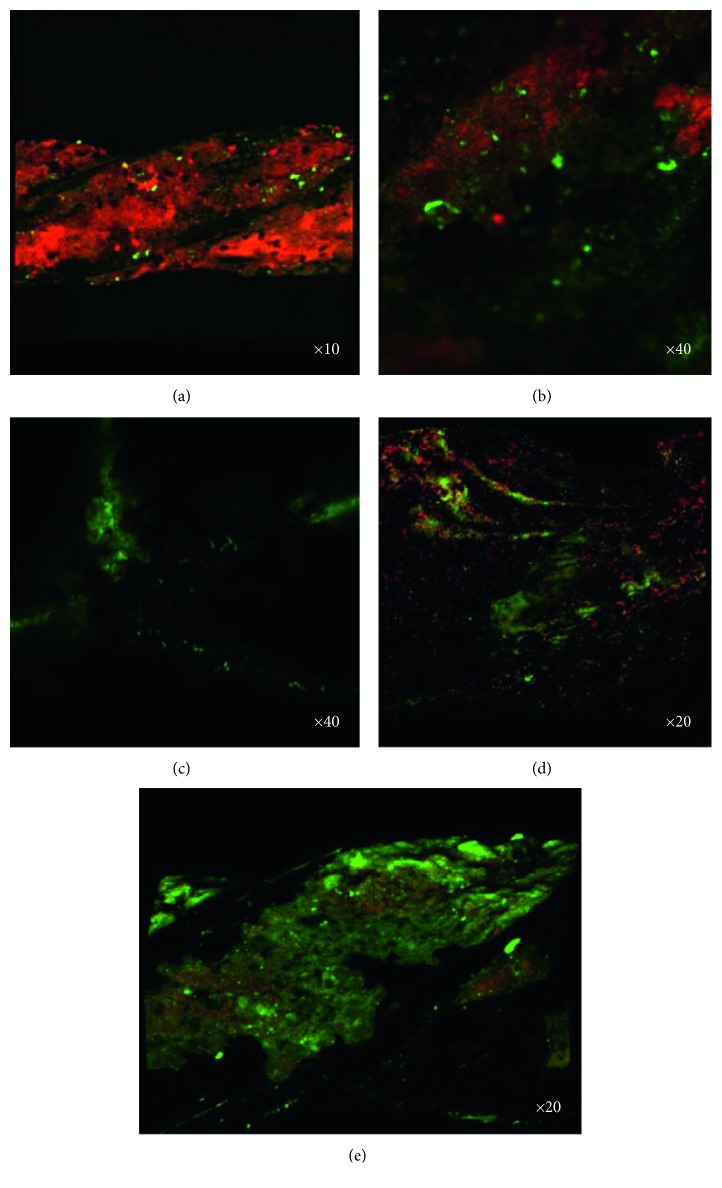
Photomicrographs of the biofilm on the surface of endodontic files, showing active cells (fluorescent green) and dead cells (fluorescent red) from the epifluorescence microscope: UEF-3 sample (a and b) with a predominance of dead cells; UEF-4 sample (c) with the absence of dead cells and with the biofilm image appearing in the shape of a Hedströem file spiral with overlapping cones; and UEF-2 (d) and UEF-1 (e) samples with a balance between dead and active cells.

The UEF-3 sample revealed a mature biofilm with a large number of dead cells deposited during the 445 days of biofilm formation and a few dispersed live cells (Figures 1(a) and 1(b)). This result was not in agreement with the negative result for the bacterial growth of the culture replica of this sample.

After the UEF-4 sample was immersed in a fresh culture medium for further 7 days, the formation of an active biofilm with a strong green fluorescence and no red fluorescence was observed (Figure 1(c)). The UEF-2 and UEF-1 samples, submitted to immersion in a new culture medium for another 14 days, revealed the presence of a biofilm composed of live and dead cells (Figures 1(d) and 1(e)).

When data from the biofilm images of the UEF-4 sample were compared with the cellular activity in the culture media for the formation of iron sulfide, the cell viability of the environmental *Desulfovibrio desulfuricans* was observed. The UEF-2 sample presented the same characteristics, with positive cultures for SRB. However, the negativity for the SRB growth in the replica of the original culture (of 445 days) may be related to the low number of viable cells. The presence of a greater number of dead cells observed in the biofilm may be related to the 14-day reimmersion time of the file, which allowed the cell growth and death of various generations during this period.

However, when analyzing the images obtained from the active biofilm of the UEF-1 sample with the negative growth for the SRB at the three different verification times, there was a reduction of growth in the culture media, demonstrating a positive cell growth of an unknown strain on this surface. Taking into account the characteristic of an anaerobic environment of the culture medium, this suggests the formation of a biofilm of an anaerobic species, optional or not, that was not isolated and of unknown species.

### 3.2. Scanning Electron Microscopy

**Figure 2:**
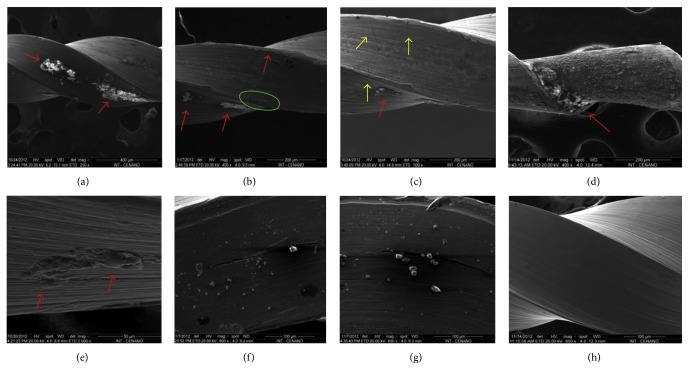
SEM analysis photomicrographs: UEF-3 sample (a, b, c) with areas suggestive of biofilm formation (red arrows), machining grooves in the metal structure (green marking (b)) and areas with cracks (yellow arrows (c)); UEF-4 sample with image suggestive of biofilm formation along the entire metal surface (d), with the highest density of the supposed biofilm indicated by the red arrow; sample UEF-5 (e), area of biofilm formation (red arrow); UEF-1 sample showing deposition of amorphous structures on the metal surface (f, g) and control sample (h).

The analysis of the UEF-2, -3, and -4 samples in SEM showed the presence of irregular and rough structures adhered along the metallic surface of the endodontic files. These structures appear as a mature calcified biofilm (Figures 2(a)–2(e)) and create an agglutination pattern or juxtaposition at the lateral cutting edge and/or in the helical channel of the endodontic files. Some images show cracks of various sizes on the metallic surface (Figure 2(c)). Due to the use of these instruments, such cracks or fracture lines may be derived from the forces generated in them during clinical use, as reported by Alapati et al. [14].

Different from the previous samples, UEF-1 presented small granular structures with a cubical shape and the presence of cracks in the metallic surface with the presence of these granular structures in the interior (Figures 2(f) and 2(g)). The images of the control file demonstrated a clean metallic surface, free of any structures like those observed in the other samples of this work. Only grooves from the machining process of the endodontic file were observed (Figure 2).

### 3.3. SEM-EDS Spot

**Figure 3:**
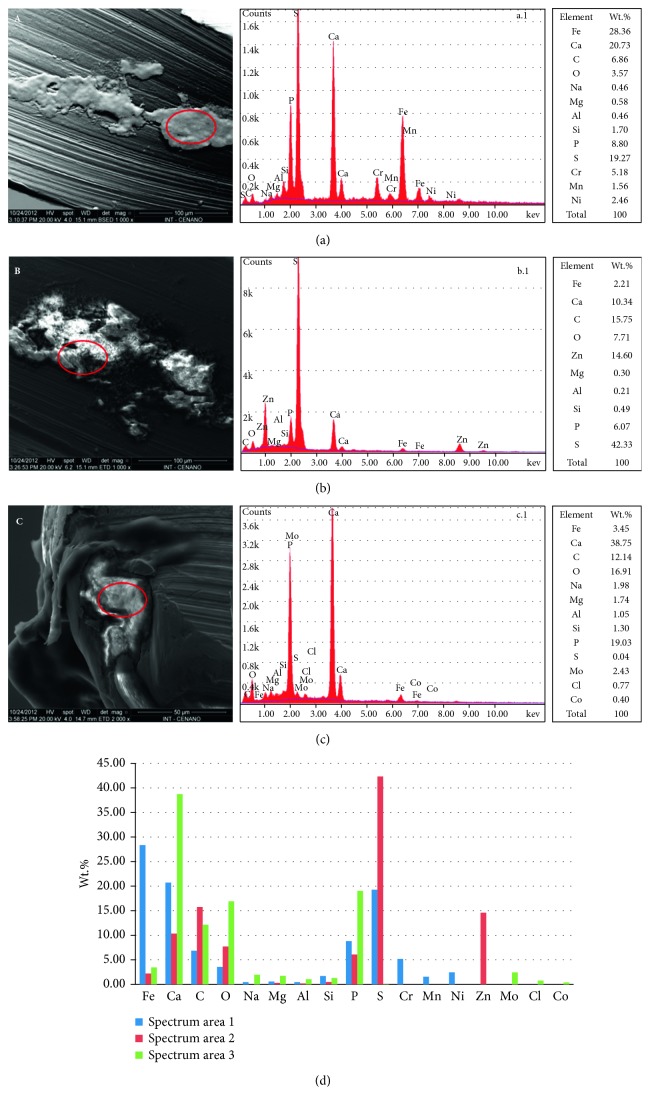
Correlation of images from SEM with the chemical spectrum obtained in the SEM-EDS spot analyses. Area of spectrum 1 (a), area of spectrum 2 (b), and area of spectrum 3 (c) of the UEF-3 sample. The spots analyzed are outlined by a red circle. Graph (d) presents a comparison between the photomicrographs (a, b, and c) of the % of each chemical element.

The three spot analyses in the areas indicative of biofilm formation on the metal surface in UEF-3 revealed a high concentration of Ca, C, O, P, and S ions, suggesting calcified biofilm formations. The presence of the S ion may be related to SRB activity, which is the main excretory element of its cell cycle (Figure 3).

Also, Na, Mg, Al, Co, Cl, and Zn were identified in addition to the chemical elements that form the metallic alloy of these files, such as Si, Mn, Cr, Ni, and Mo, present in a lower concentration in the chemical spectra. This suggests a possible corrosive action of SRB on the endodontic file, transferring such elements to the mature biofilm; however, this spot analysis has a limited beam depth.

The elements S, C, and P extrapolated the normal values in a metallic alloy, as reported by Heggendorn et al. [15], which are 0.001% S, 0.079% C, and 0.017% P. These data and the presence of Ca suggest that this is a mature SRB biofilm, while the presence of O may be related to the oxidation of the metallic surface and/or bacterial activity.

### 3.4. SEM-EDS Quantitative

The average of the quantitative spectra of each UEF with a biofilm formation on the metal surface showed that there was a reduction of the Fe, Cr, and Ni metal alloying elements when compared with the control sample (62.58% Fe, 15.51% Cr, and 6.60% Ni). The averages of the files were UEF-2 (56.34% Fe, 13.76% Cr, and 5.67% Ni), UEF-3 (54.75% Fe, 13.43% Cr, and 5.75% Ni), and UEF-4 (32.87% Fe, 8.87% Cr, and 3.75% Ni). While for the elements Ca and P, UEF-4 (6.81% Ca and 6.45% P) had the highest quantities of these elements compared with the others: UEF-2 (2.01% Ca and 1.47% P) and UEF-3 (1.09% Ca and 0.99% P).

**Figure 4:**
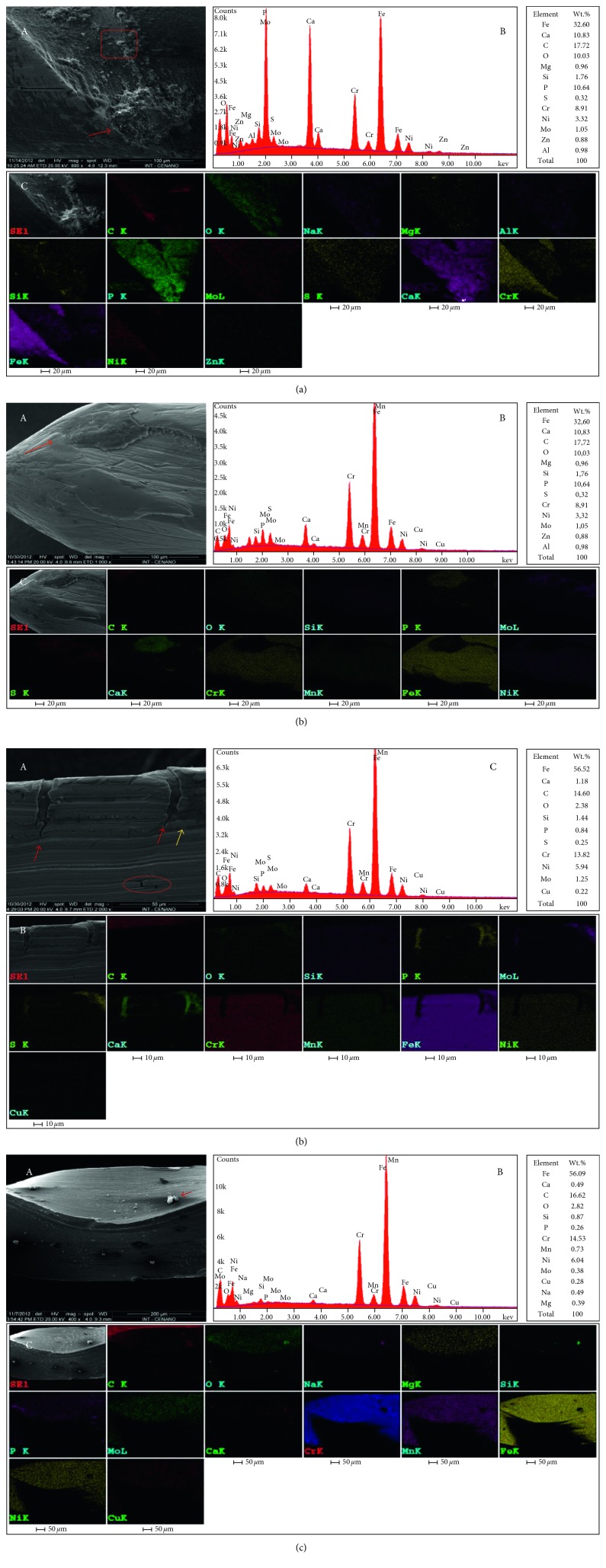
Composition of the spectra of UEF-4 (a), UEF-2 (b and c), and UEF-1 (d). Correlation of images obtained in the SEM-EDS mode (a-A and d-A) and SEM-EDS (b-A and c-A) with the chemical spectrum obtained in the SEM-EDS mode (B) X-ray mapping (C) demonstrating the spatial distribution of the chemical elements. The images reveal the largest areas of mature biofilm formation (red arrows), the area marked by red square indicates the biofilm interface and metallic surface (a-A), area suggestive of corrosion (yellow arrow) (c-A), and area suggestive of pits (marked by the red circle) (c-A). However, the composition of (d) show a point with high concentration of (C) O, Na, and Si.

The elements C and O also showed a higher percentage presence in the files with biofilm formation, UEF-4 (25.42% C and 7.76% O), UEF-3 (15.42% C and 3.28% O), and UEF-2 (12.75% C and 2.84% O). Na and Mg presented a similar pattern, with a higher concentration in UEF-4 (1.26% Na and 0.75% Mg) and UEF-3 (0.45% and 0.65% Mg) for the samples that are positive for the SRB biofilm formation. The UEF-1 sample had an unknown biofilm that was less extensive and presented 0.16% Na and 0.11% Mg. The UEF-1 sample (61.09% Fe, 15.53% Cr, 6.77% Ni, 0.19% P, and 1.95% O), negative to SRB formation, showed similar spectra to the control sample in relation to Fe, Cr, Ni, P (0.09%), and O (2.09%). The elements C and Ca were higher in UEF-1 (11.93% C and 0.37% Ca) when compared with UEF-control (9.53% C and Ca absent) (Figure 4).

The strongest indication of SRB activity is possibly related to the presence of S; in this work, the highest percentages of S were found in samples considered positive for SRB: UEF-4 (0.68%), UEF-2 (0.49%), and UEF-3 (0, 10%) and the lowest in samples considered negative for SRB: UEF-1 (0.04%) and control (0.07%) as shown in Figure 4. The analyzed spectra of UEF-1, negative to SRB growth in the stages of bacterial growth analysis, showed S in the quantitative SEM-EDS mode in only one chemical spectrum, while UEF-3, UEF-4, and UEF-2 presented S in all spectra analyzed. In these analyses, the UEF-3 sample suggested the formation of a mature, sessile biofilm. Thus, the presence of S in this biofilm could have already been reduced when related to the presence of SRB, since the number of dead cells was higher than the number of living cells in the epifluorescence microscopy analysis. However, the samples that presented the formation of an active biofilm showed higher levels of S, as seen in the UEF-4 sample, which showed a reactivated biofilm due to the extended seven-day culture, followed by the UEF-2 sample, with a reactivated biofilm coming from an extended 14-day period. In addition to S, the UEF-4 sample also showed the highest concentrations of Ca, P, and C, which are the bacterial biofilm-forming elements.

### 3.5. SEM-EDS X-Ray Mapping

The quantitative spectra and X-ray mapping of UEF-3 revealed the similarities in the presence of the chemical elements observed in the SEM-EDS spot analysis, except Zn, Cl, and Co (present in the spot spectra) and including Cu in three of the four X-ray mapping analyses. The concentration profile of the chemical elements of this analysis is different due to the percentage concentration of the mass from the first quantitative SEM-EDS which was spot, and this analysis is of an area corresponding to the total image generated in the SEM.

Four X-ray mappings were performed for UEF-3 and UEF-4 and two for UEF-2, which showed areas with high concentrations of P, Ca, and C followed or not by S, Na, O, and Si in areas suggestive of biofilm formation.

**Figure 5:**
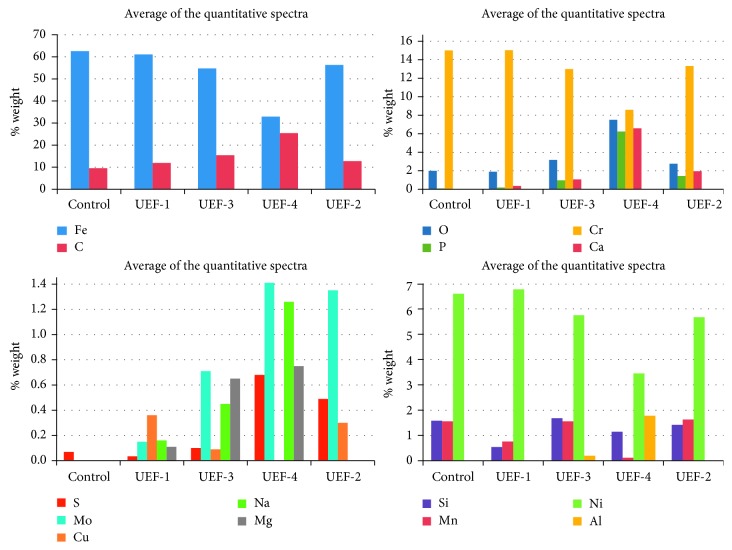
Mean of the quantitative spectra of the X-ray mapping of the samples.

In all the areas suggestive of biofilm formation, there was an absence or reduction in the concentrations of Cr, Mn, Fe, and Ni (Figure 5). The 3 X-ray mappings performed on UEF-1, which did not show SRB growth at any stage, presented particles of different sizes, adhering to a smaller length and area of the metallic surface of the file when compared to that of other samples. These structures in UEF-1 presented distinct differences in size and shape when compared to biofilms of the SRB-positive samples.

In Figure 5, the X-ray mapping of UEF-4 presented an area with a very clear delimitation of the biofilm with the metal surface of the endodontic file at a level below the supposed biofilm. The presence of the element S indicates a higher concentration of the biofilm. The area of the metal surface of the file without biofilm formation showed high concentrations of Fe, Cr, and Ni, an absence of P and Ca, and low concentrations of O and Na (Figure 5).

In UEF-5 (Figure 5), cracks in the metal surface with high concentrations of P, Ca, and S, and an absence of Cr, Mn, F, and Ni could be seen. The region marked by a red circle appears to show corrosion pits with the same characteristics as the X-ray mapping, except for the presence and absence of S and Mn, respectively.

UEF-1 showed a surface covered with particles with high concentrations of C, O, Na, and Si and the absence of Fe, Ni, Mn, and Cr (Figure 5).

When comparing the images of the X-ray mappings, a difference in the metal surface of the SRB-positive samples (UEF-2, UEF-3, and UEF-4) was evident compared with the metal surface of the UEF-1 sample that was negative for SRB growth, but with the growth of an unknown anaerobic bacterial strain. The most important feature was the deposition of the associated Ca and P elements or ions and in some images with C on the metallic surface of the SRB-positive samples, which suggests the formation of a biofilm on the metallic surface with different sizes and shapes, which may extend over a large part of the surface or in areas of cracks. In the negative SRB sample (UEF-1), these deposits had the form of small cube-like granules with a very weak presence on the metallic surface.

## 4. Discussion

Okabe et al. [16] reported the use of CLSM with the fluorophore TRITC to analyze the spatial distribution of SRB in 40-day-old aerobic biofilms. The authors demonstrated the presence of SRB and mineral compounds. They described a structured biofilm surface similar to the ones presented in this study, forming microbial aggregates and interstitial voids. The associated use of CLSM and SEM was described by Dunsmore et al. [17] and Liu et al. [18] to observe SRB biofilm. For Liu et al. [18], the use of SEM determined the distribution and morphology of the biofilm, and it was possible to correlate these results with the fully hydrated biofilm images obtained in the CLSM. As in our results, the difficulty in differentiating some areas of pits on the surface of endodontic files observed in SEM was also demonstrated by Marending et al. [19].

White and Gadd [20] characterized the presence of P, Ca, S, Fe, and Cu in 7, 14, and 21-day-old SRB cultures, and they identified a nonuniform distribution of Cu and S in the biofilms. Subsequently, Remoundaki et al. [21] revealed high concentrations of O, Mg, P, S, Zn, Fe, C, and N in SEM-EDS analyses. To these authors, the spectrum of the bacterial population showed a high concentration of S, Zn, and O followed by the presence of P, Mg, Cl, Fe, Ca, C, and N. Comparing their data with the UEF-3 spot spectra and the other spectra, all the elements are in agreement with our analyses except N, which was absent. However, Zn was present in the UEF-4 sample. The presence of a broader chemical spectrum in the analysis of the UEF-3 sample and in the other samples, in comparison to the results observed by these authors, is due to the presence of the endodontic files releasing ions from their metallic alloy in the medium, thus allowing sequestration of these elements by the biofilm forming on the surface of the endodontic file. Also, Chen et al. [22] reported the absorption of metal ions by the EPS until reaching a balance with the medium. Brown et al. [23] correlated the identification of P to the nucleic acids and phospholipids of the biofilm cells in the SEM-EDS as well as Mg and Ca to cytoplasmic electrolytes. This may clarify the presence of these elements in samples UEF-1, -2, -3, and -4.

There have also been reports of O_2_ in aerobic biofilms of SRB, varying from very low to zero in the centers of microcolonies that form the biofilms, besides the presence of Mg dissolved under areas of biofilm colonization [16, 24, 25]. The results of these authors are in accordance with the X-ray mappings of the UEF-2, -3, and -4 samples with regular distribution of the Mg and O ions on the metallic surface and concentrations of the O ions in some areas, suggestive of biofilm formation. Also, the analysis of the mean values of these two chemical elements (Mg and O) in the UEF-4 sample, with reactivation of the seven-day biofilm, showed that UEF-4 presented the highest level of O (7.76% O), followed by UEF-3 (3.28% O), UEF-2 (2.84% O), and UEF-1 (1.95% O) negative for the growth of SRB and UEF-control (2.05% O). This may have been due to the fact that the samples with higher levels of the O ion are those that were inoculated with SRB, which may be related to the confinement of this ion in the biofilm or to the corrosion process. Lens et al. [26] identified in biofilms of 150 days the presence of PO^3−^_4_−P composing the biomass. These results are in agreement with the spot analysis of the UEF-3 sample, which presented the highest O levels, with a mean of 9.39%. However, the presence of the Mg ion (UEF-4 (0.75% Mg), UEF-3 (0.65% Mg), UEF-1 (0.11% Mg), and UEF-2 and UEF-control (absence)) could not be related to the biofilm analysis.

Gu et al. [27] listed the ions present in a microenvironment where SRB was present: Fe^2+^, SO_4_^2−^, OH^−^, and H_2_S, all of which are involved in the corrosion process. Videla et al. [28, 29] showed the presence of SO_4_^2−^, Cl^−^, S^0^, or S_2_O_3_^2−^ ions in environments with biotic corrosion, with the presence of SRB and abiotic, without the involvement of the bacteria. Also, these authors reported the presence of FeS and FeS_2_ in both the biotic and abiotic conditions in the outermost layers of the biofilms. In addition to the S, O, and Fe ions found in association in most of the GLU samples, Cl was found only in the UEF-3 sample in the spot spectrum of area 3 (0.77% CI). Dunsmore et al. [17] and Yuan et al. [30] described a metal surface covered by dense and porous SRB biofilm clusters, which suggest localized attacks by aggressive Cl^−^ and S^2−^ ions, leading to the onset of corrosion. This description coincides with the images presented in our SEM evaluation (Figures 2 and 5) with localized formations of calcified areas and in some images where it was possible to see areas of corrosion.

Purish et al. [31] analyzed a 90-day-old *Desulfovibrio* biofilm on a metallic surface. These authors demonstrated the accumulation of hydrogen sulfide in the biofilm and polysaccharides and other carbons in the composition of the biofilm matrix. These polysaccharides are capable of binding to metal ions and sulfides in a matrix [32], according to the chemical mapping analyses that verified C and S in areas suggestive of biofilm. Previously, Lopes et al. [32] reported the presence of S on the surface of SS 304 stainless-steel coupons in *Desulfovibrio desulfuricans* cultures, with higher concentrations of S under anaerobic conditions, with between 23 and 34 days of culture. The presence of H_2_S with large amounts of Fe and Mn may react and form insoluble metallic sulfide, S, and polysulfides [33], which may be correlated to the deposits that form the biofilm matrices found in the samples before pickling. The identification of a large number of dead cells in the epifluorescence microscopy of the UEF-3 sample may be correlated with the fact that a part of the bacterial population is inactivated due to encapsulation by the iron sulfide present in the medium [21]. Considering that this sample was maintained for a long period of culture without renewal of the culture medium may have led to a saturation of the medium by iron sulfide.

The distribution and immobilization of inorganic substances in the biofilm depend on the properties of their absorption capacity, as determined by the pH, as well as the type and concentration of the binders present in the biofilm matrix [34]. In general, a low pH will result in a release of ions from a bound state while high pH tends to favor chelation [34]. However, in our analyses, the pH at all times was close to neutral, which suggested that there was no interference due to pH in the chemical pattern found in the biofilms of the metallic files.

The biocorrosion rate profiles presented here were inexpressive when correlated with the long immersion times of the endodontic files. However, Isa et al. [35] demonstrated that the highest activity of SRB in anaerobic reactors was between 11 and 24 days, and the production of S decreased between 54 and 63 days. This fact can be explained by the supersaturation of iron sulfide in the medium, as described by Jhobalia et al. [36], who confirmed the sudden drop of corrosion in steel coupons when the solution was supersaturated with FeS_2_, which then remained stable. Throughout the tests, the authors reported that increased sulfide concentration decreased the SRB growth rate and corrosion rate. Lopes et al. [32] related the concentration of nickel to the viability of *Desulfovibrio desulfuricans*. High nickel concentrations reduced the rate of cell duplication while low levels were shown to be positive for cell growth [32]. However, chromium, although present in the metallic surfaces, did not present an effect on the time of duplication of the bacteria in the studies by Lopes et al. [32]; also, molybdenum has been indicated for retarding the cell growth of *Desulfovibrio desulfuricans* [37]. In the analyses presented in our work, Ni and Cr were present in all the samples, and the highest concentrations were seen in the UEF-control and UEF-1 samples.

The importance of demonstrating the presence of the biofilm lies in the fact that the formation of the SRB biofilm on the metal surface will directly influence the corrosion rate, altering the transport of chemical elements favorably or unfavorably, facilitating the removal of the protective film on the metal surface and inducing differential aeration as a consequence of the irregular biofilm distribution [25]. The choice of testing used endodontic files is justified by the structural modifications on the metallic surface, which only such instruments would possess, since characteristics such as the roughness and porosity of their surfaces are fundamental for the process of allowing bacteria to adhere to them [38].

## 5. Conclusions

The chemical elements shown to be present by SEM-EDS analyses suggest the presence of an irregular SRB biofilm on the endodontic files studied. However, the EDS does not provide the oxidation state of the chemical elements identified, which would be important for an effective demonstration of mineral accumulation in the biofilm. New investigations into the formation of biofilms on steels are important, since they represent the initial stage in microbiologically influenced corrosion (MIC).

Thus, our results here suggest the formation of SRB biofilms on used endodontic files, with characteristics that may contribute to the biocorrosion of these files, and these results may also provide complementary data for a biopharmaceutical under development that assists in the removal of fractured endodontic files inside root channels.

## Figures and Tables

**Table 1:** Description of the immersion test for the used endodontic files.

Used endodontic files (UEFs)	Culture medium	Bacteria inoculated	File	SRB growth after 30/445 days
UEF-1	Postgate C culture medium with 7.0 g/l of agar-agar (vehicle I)	*Desulfovibrio desulfuricans* (oral)	Kerr #40	Reduction/reduction
UEF-2	Modified Postgate E culture medium	*Desulfovibrio fairfieldensis* (association)	Kerr #25	Positive/positive
UEF-3	Modified Postgate E culture medium	*Desulfovibrio desulfuricans* (environmental)	Kerr #20	Positive/positive
UEF-4	Modified postgate E culture medium	*Desulfovibrio desulfuricans* (environmental)	Hedströem #25	Positive/positive

**Table 2:** Distribution of the samples in relation to the methodological applications of microbiological evaluation.

Sample	Immersion time (days)	Replica of original culture	SRB growth after reimmersion in culture medium	Replica of reimmersion culture
UEF-3	445	Negative	Not performed	Not performed
UEF-4	452	Positive	Positive	Positive
UEF-2	459	Negative	Positive	Positive
UEF-1	459	Negative	Reduction of culture medium	Negative

**Table 3:** Measurement of pH at different cell culture times and cell viability of replicas from the different culture times.

		Postgate C culture medium	Modified Postgate E culture medium		
Time		*Desulfovibrio desulfuricans* (oral)	*Desulfovibrio desulfuricans* (environmental)		*Desulfovibrio fairfieldensis* (association)
		UEF-1	UEF-3	UEF-4	UEF-2
pH/cell growth	Culture 445 days	pH 7/reduction	pH 7/positive	pH 7/positive	pH 7/positive
	Cultivation 7 days (maintenance of 445-day biofilm)	—	—	7/positive	—
	Cultivation 14 days (maintenance of 445-day biofilm)	pH 7/reduction	—	—	pH 7/positive
Replica	Replica of original culture (445 days)	Negative	Negative	Positive	Negative
	Replica of samples of reimmersion culture	Negative	—	Positive	Positive
